# Continuing immune checkpoint inhibitors beyond progression versus switching to non-ICI therapy in advanced gastric cancer: a real-world study

**DOI:** 10.3389/fonc.2026.1798205

**Published:** 2026-06-03

**Authors:** Guangxi Wang, Xiaonan Lu, Yunmei Wang, Xiang Wang, Ruoxuan Wang, Jinyu Jia

**Affiliations:** 1 Department of Medical Oncology, Shaanxi Provincial Cancer Hospital, Xi’an, China; 2 Department of Ultrasound, The First Affiliated Hospital of Air Force Medical University, Xi’an, China

**Keywords:** advanced gastric cancer, efficacy, immune checkpoint inhibitor, real-world study, safety, treatment beyond progression

## Abstract

**Background:**

The optimal second-line therapy for advanced gastric/gastroesophageal junction cancer (GC/GEJC) following progression on first-line immuno-chemotherapy remains undefined. This study aimed to compare the efficacy and safety of continuing an immune checkpoint inhibitor beyond progression (CIBP) versus switching to a non-ICI regimen (non-CIBP).

**Methods:**

In this single-center, retrospective study, we enrolled 169 patients with GC/GEJC who progressed after first-line ICI plus chemotherapy. Based on the second-line therapeutic decision, patients were categorized into either the CIBP group (n=120; to continue ICI with or without combined therapy) or the non-CIBP group (n=49; to switch to a non-ICI regimen). The primary endpoints were progression-free survival (PFS) and overall survival (OS).

**Results:**

The CIBP group demonstrated significantly improved median PFS (4.4 vs. 3.0 months; hazard ratio [HR]=0.47, 95% CI: 0.33-0.67; p<0.001) and median OS (9.5 vs. 6.4 months; HR = 0.51, 95% CI: 0.34-0.77; p=0.0012) compared to the non-CIBP group. The survival benefit of CIBP remained robust after multivariable adjustment (PFS HR = 0.54, p=0.003; OS HR = 0.65, p=0.044) and propensity score matching. Subgroup analyses revealed that patients with PD-L1 expression ≥1%, those who achieved an objective response to first-line therapy, or those with a first-line PFS ≥6 months derived the greatest benefit from CIBP. The safety profile was manageable and comparable between the two groups.

**Conclusion:**

In this real-world cohort, continuing ICI beyond progression was associated with significantly superior survival outcomes compared to switching to non-ICI regimens, with a manageable safety profile. CIBP represents a valid and promising second-line strategy for selected patients with advanced GC/GEJC following first-line immuno-chemotherapy failure.

## 1 Introduction

Gastric cancer (GC) is a major global health concern, characterized by high incidence and mortality. The 2020 GLOBOCAN report documented approximately 1.09 million new cases and 769, 000 deaths annually, highlighting a substantial disease burden that is particularly pronounced in East Asia (1, 2). Patients with advanced or metastatic disease face a poor prognosis, with a 5-year survival rate below 20%. Although chemotherapy has long been the first-line backbone, its efficacy is limited, underscoring the need for more effective treatments (3).

The treatment landscape for advanced GC has been transformed by immune checkpoint inhibitors (ICIs). By targeting PD-1/PD-L1 pathways, ICIs reverse T-cell suppression and reinvigorate anti-tumor immunity (4). This shift is supported by landmark phase III trials, including CheckMate-649, which established nivolumab plus chemotherapy as a global standard in HER2-negative GC (5), and KEYNOTE-811, which confirmed the efficacy of pembrolizumab in HER2-positive disease (6). Positive results from Chinese trials such as ORIENT-16 and RATIONALE-305 further solidified the role of sintilimab and tislelizumab (7, 8), making PD-1 inhibitor–based combinations a cornerstone of first-line therapy.

However, the success of first-line immuno-chemotherapy has created a new clinical dilemma regarding the optimal second-line strategy after disease progression. The pivotal trials that established first-line standards were not designed to inform subsequent therapy, creating a significant evidence gap in major clinical guidelines (6, 8). This lack of high-level evidence is reflected in the poor outcomes of existing second-line options, as demonstrated by a recent meta-analysis reporting benchmark median overall survival and progression-free survival of only 7.9 and 3.5 months, respectively (9). Consequently, the choice between continuing an immune checkpoint inhibitor beyond progression (CIBP) or switching to a non-ICI regimen remains highly controversial and contributes to heterogeneous real-world practice. To directly address this critical gap, we conducted this real-world study aiming to compare the efficacy of CIBP versus non-CIBP regimens in patients with advanced gastric cancer after first-line immuno-chemotherapy failure, with overall survival and progression-free survival as primary endpoints. Comprehensive subgroup analyses were also performed to identify potential beneficiaries of CIBP. We anticipate our findings will provide robust evidence to guide therapeutic selection and help optimize patient stratification for future prospective trials.

## 2 Methods

### 2.1 Study design and participants

**Figure 1:**
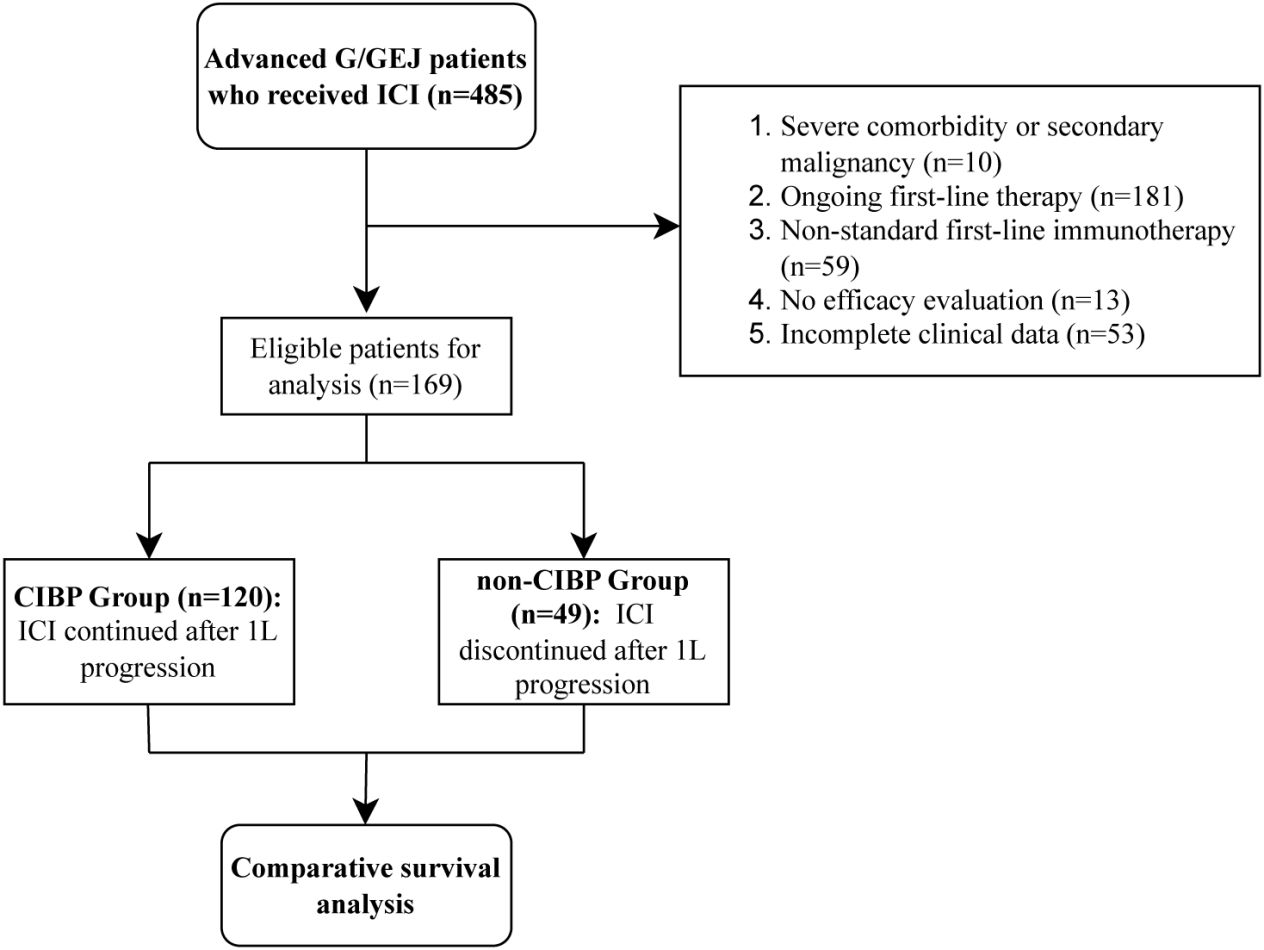
Patient selection flowchart. G/GEJ, gastric or gastroesophageal junction; ICI, immune checkpoint inhibitor; CIBP, continuing immune checkpoint inhibitor beyond progression. This flowchart illustrates the screening process and final composition of the study cohort. A total of 485 patients with advanced G/GEJ cancer who received first-line immunotherapy were initially screened. After applying the exclusion criteria, 169 eligible patients were included in the final analysis and categorized into two groups based on their second-line treatment strategy: the CIBP group (n=120) and the non-CIBP group (n=49).

This was a single-center, real-world retrospective study conducted at Shaanxi Provincial Cancer Hospital. We screened patients with advanced GC/GEJC who progressed after first-line ICI plus chemotherapy (with or without anti-HER2 therapy) and received second-line therapy between January 1, 2020, and October 31, 2025. Key inclusion criteria were: histological confirmation and having received at least one cycle of second-line therapy with efficacy evaluation. No restrictions were placed on HER2 status or baseline ECOG score. Exclusion criteria included: treatment intolerance, severe autoimmune diseases, multiple primary cancers, or incomplete clinical data. The detailed patient selection process is illustrated in the study flowchart (**Figure 1**). This study was approved by the Ethics Committee of Shaanxi Provincial Cancer Hospital (Approval No. YLS [2025] No. 131), and the requirement for informed consent was waived.

### 2.2 Treatment regimens

Based on the second-line treatment strategy, all enrolled patients were categorized into two groups: CIBP group (Continuing ICI Beyond Progression) and Non-CIBP group. In the CIBP group, patients continued to receive an immune checkpoint inhibitor in the second-line setting, with or without other systemic therapies. Specific ICI-based combinations included: chemotherapy, anti-angiogenic agents, antibody-drug conjugates (ADCs), or anti-HER2 therapy. In the Non-CIBP group, patients did not receive any ICI in the second-line setting. The treatment regimens included, but were not limited to: chemotherapy alone, chemotherapy combined with anti-angiogenics, anti-angiogenics alone, ADCs, or anti-HER2 therapy. The specific chemotherapeutic regimens (e.g., platinum-based, fluoropyrimidine-based, or taxane-based combinations), anti-angiogenic agents (e.g., Apatinib, Anlotinib, Ramucirumab), and ADC drugs (e.g., Disitamab vedotin) used in both groups were in accordance with standard clinical practice in China. For patients with HER2-positive tumors, anti-HER2 therapy (e.g., Trastuzumab, Trastuzumab emtansine) could be incorporated into the treatment regimen of either group, as clinically indicated.

### 2.3 Outcomes

The primary endpoint of this study was progression-free survival (PFS), defined as the time from the initiation of second-line therapy to disease progression or death from any cause. Secondary endpoints included: overall survival (OS), defined as the time from the initiation of second-line therapy to death from any cause; objective response rate (ORR), defined as the proportion of patients achieving a complete response (CR) or partial response (PR); and disease control rate (DCR), defined as the proportion of patients with a best overall response of CR, PR, or stable disease (SD). Tumor response was typically assessed according to the Response Evaluation Criteria in Solid Tumors (RECIST) version 1.1. The safety endpoint included the evaluation of adverse events (AEs). All AEs were monitored and graded according to the National Cancer Institute Common Terminology Criteria for Adverse Events (CTCAE), version 5.0.

### 2.4 Statistical analysis

All statistical analyses were performed using R software (version 4.5.1). Patient baseline characteristics were summarized using descriptive statistics. Categorical variables were presented as frequencies (percentages) and compared between groups using the Chi-square test or Fisher’s exact test. Continuous variables (e.g., age) were presented as median (interquartile range) based on their distribution, and compared using the Student’s t-test or Mann-Whitney U test. Survival analyses were conducted using the Kaplan-Meier method, with group comparisons performed by the Log-rank test. To evaluate the independent impact of treatment beyond progression (CIBP) on outcomes, univariate Cox proportional hazards regression analyses were first performed to identify prognostic factors associated with overall survival (OS) and progression-free survival (PFS). Subsequently, the treatment group (CIBP vs. non-CIBP) and all variables showing significance in the univariate analysis (typically at P < 0.1) were included in a multivariate Cox proportional hazards model to calculate adjusted hazard ratios (HRs) with 95% confidence intervals (CIs). Subgroup analyses were performed to assess the consistency of the treatment effect across patients with different baseline characteristics, visualized using forest plots. The presence of effect modification was tested by incorporating an interaction term into the Cox model and using the likelihood ratio test to assess for statistically significant interactions.

To enhance the robustness of the findings, a sensitivity analysis was conducted using propensity score matching (PSM). The matching incorporated seven clinically relevant baseline variables: age (≥65 vs <65 years), ECOG performance status (≥2 vs 0-1), peritoneal metastasis, metastatic burden (number of metastatic sites), histology type (non-intestinal vs intestinal), PD-L1 expression (≥1% vs <1%), and first-line progression-free survival (≥6 vs <6 months). A 1:1 nearest-neighbor matching algorithm was employed with a caliper width of 0.1 to optimize inter-group balance. Survival outcomes in the propensity score-matched cohort were evaluated using Kaplan-Meier curves with the log-rank test and Cox proportional hazards models.

To address potential immortal time bias, we performed a 2−month landmark analysis as a sensitivity analysis. Detailed methods and results are provided in the **Supplementary Material**.

A two-sided P-value < 0.05 was considered statistically significant for all tests.

## 3 Results

### 3.1 Baseline characteristics

A total of 485 patients with advanced G/GEJ cancer who received first-line immunotherapy were initially screened. After applying the exclusion criteria, 169 patients who received second-line therapy were ultimately included in this analysis. These patients were categorized into two groups based on their second-line treatment strategy: the CIBP group (Continuing ICI Beyond Progression, n=120), who received an ICI ± chemotherapy/anti-angiogenic therapy/ADC, and the non-CIBP group (n=49), who received chemotherapy, anti-angiogenic therapy, or ADC without an ICI. The baseline demographic and clinical characteristics of the entire cohort and the two groups are summarized in **Table 1**.

**Table 1:** Baseline demographic and clinical characteristics.

Variable	Overall (N = 169)^1^	CIBP (N = 120)^1^	Non-CIBP (N = 49)^1^	P
Age (years)	63 (56, 70)	62 (56, 69)	65 (58, 71)	0.520
Age group				0.417
≥ 65	78 (46.2%)	53 (44.2%)	25 (51.0%)	
≤ 65	91 (53.8%)	67 (55.8%)	24 (49.0%)	
Sex				0.458
Female	55 (32.5%)	37 (30.8%)	18 (36.7%)	
Male	114 (67.5%)	83 (69.2%)	31 (63.3%)	
ECOG PS				0.056
0~1	121 (71.6%)	91 (75.8%)	30 (61.2%)	
≥ 2	48 (28.4%)	29 (24.2%)	19 (38.8%)	
Histological type				0.133
Intestinal	72 (42.6%)	47 (39.2%)	25 (51.0%)	
Diffuse	67 (39.6%)	53 (43.8%)	14 (29.2%)	
Poorly cohesive	50 (29.6%)	41 (34.2%)	9 (18.4%)	
Signet ring cell	17 (10.1%)	12 (10.0%)	5 (10.2%)	
Mixed	12 (7.1%)	10 (8.3%)	2 (4.1%)	
Other/Unclassified	18 (10.7%)	10 (8.3%)	8 (16.3%)	
Primary site				0.269
G	114 (67.5%)	84 (70.0%)	30 (61.2%)	
GEJ	55 (32.5%)	36 (30.0%)	19 (38.8%)	
TNM stage				0.676
I-II	3 (1.8%)	3 (2.5%)	0 (0.0%)	
III	24 (14.2%)	18 (15.0%)	6 (12.2%)	
IV	142 (84.0%)	99 (82.5%)	43 (87.8%)	
Metastasis Sites				
Liver	59 (34.9%)	38 (31.7%)	21 (42.9%)	0.166
Lung	31 (18.3%)	20 (16.7%)	11 (22.4%)	0.378
Brain	6 (3.6%)	3 (2.5%)	3 (6.1%)	0.248
Bone	20 (11.8%)	15 (12.5%)	5 (10.2%)	0.675
Peritoneal	50 (29.6%)	36 (30.0%)	14 (28.6%)	0.854
Pelvic*	17 (10.1%)	9 (7.5%)	8 (16.3%)	0.083
Lymph node	79 (46.7%)	63 (52.5%)	16 (32.7%)	0.019
Number of metastatic sites				0.901
< 3	115 (68.0%)	82 (68.3%)	33 (67.3%)	
≥ 3	54 (32.0%)	38 (31.7%)	16 (32.7%)	
PD-L1 expression^‡^				0.079
< 1%	59 (34.9%)	39 (32.5%)	20 (40.8%)	
1~5%	30 (17.8%)	24 (20.0%)	6 (12.2%)	
> 5%	40 (23.7%)	33 (27.5%)	7 (14.3%)	
NA	40 (23.7%)	24 (20.0%)	16 (32.7%)	
HER2 status				0.204
0	60 (35.5%)	39 (32.5%)	21 (42.9%)	
1+/2+ ISH-	70 (41.4%)	53 (44.2%)	17 (34.7%)	
2+ ISH+/3+	19 (11.2%)	16 (13.3%)	3 (6.1%)	
NA	20 (11.8%)	12 (10.0%)	8 (16.3%)	
MMR status				0.163
dMMR/MSI-H	4 (2.4%)	4 (3.3%)	0 (0.0%)	
pMMR/MSS	141 (83.4%)	102 (85.0%)	39 (79.6%)	
NA	24 (14.2%)	14 (11.7%)	10 (20.4%)	
Prior treatments^†^				
Surgery	60 (35.5%)	44 (36.7%)	16 (32.7%)	0.621
Radiotherapy	13 (7.7%)	9 (7.5%)	4 (8.2%)	0.883
Neoadjuvant CT	14 (8.3%)	9 (7.5%)	5 (10.2%)	0.563
Adjuvant CT	46 (27.2%)	33 (27.5%)	13 (26.5%)	0.898
Anti-HER2	17 (10.1%)	13 (10.8%)	4 (8.2%)	0.601
First-line ICI				0.889
Sintilimab	86 (50.9%)	62 (51.7%)	24 (49.0%)	
Tislelizumab	42 (24.9%)	28 (23.3%)	14 (28.6%)	
Camrelizumab	20 (11.8%)	15 (12.5%)	5 (10.2%)	
Pembrolizumab	7 (4.1%)	6 (5.0%)	1 (2.0%)	
Nivolumab	9 (5.3%)	6 (5.0%)	3 (6.1%)	
Toripalimab	5 (3.0%)	3 (2.5%)	2 (4.1%)	
PFS1 > 6 months	94 (55.6%)	67 (55.8%)	27 (55.1%)	0.931

CIBP, continuing immune checkpoint inhibitor beyond progression; CT, chemotherapy; ECOG PS, Eastern Cooperative Oncology Group performance status; G, gastric; GEJ, gastroesophageal junction; ICI, immune checkpoint inhibitor; IQR, interquartile range; ISH, *in situ* hybridization; dMMR, deficient mismatch repair; pMMR, proficient mismatch repair; MSI-H, microsatellite instability-high; MSS, microsatellite stable; NA, not available.

1.Data are presented as median (IQR) or n (%). *Pelvic metastases include soft tissue, ovarian, fallopian tube, and other pelvic organ involvement.†Any prior treatments received before second-line therapy.‡PD-L1 expression assessed by combined positive score (CPS).P values were derived from the χ² test, Fisher’s exact test, Student’s t-test, or Mann-Whitney U test, as appropriate.

The median age of the overall population was 63 years (IQR: 56-70), with 114 (67.5%) being male. Most patients had an ECOG PS of 0-1 (71.6%), primary gastric cancer (67.5%), and stage IV disease (84.0%). The baseline characteristics were generally well-balanced between the CIBP and non-CIBP groups. Apart from a higher incidence of lymph node metastasis in the CIBP group (52.5% vs. 32.7%, p=0.019), other key prognostic variables—including age, sex, ECOG PS, TNM stage, number of metastatic sites, PD-L1 expression, HER2 status, and the duration of first-line treatment benefit (PFS1 > 6 months)—all showed no statistically significant differences (all p > 0.05), indicating excellent comparability between the two groups for subsequent efficacy analyses.

### 3.2 Treatment efficacy

The antitumor activity of second-line regimens is summarized in **Table 2**. The objective response rate (ORR) was numerically higher in the CIBP group compared to the non-CIBP group (11.7% vs. 4.1%), though this difference was not statistically significant (p=0.215). In contrast, the disease control rate (DCR), representing the proportion of patients achieving at least stable disease, was significantly improved in the CIBP group (72.5% vs. 55.1%, p=0.045).

**Table 2:** Antitumor activity of second-line therapy by treatment group.

Best overall response, n (%)	Total (N = 169)	CIBP (N = 120)	Non-CIBP (N = 49)	P value
CR	–	–	–	
PR	16 (9.5)	14 (11.7)	2 (4.1)	
SD	98 (58.0)	73 (60.8)	25 (51.0)	
PD	55 (32.5)	33 (27.5)	22 (44.9)	
ORR	16 (9.5)	14 (11.7)	2 (4.1)	**0.215**
DCR	114 (67.5)	87 (72.5)	27 (55.1)	**0.045**

CIBP, continuing immune checkpoint inhibitor beyond progression; CR, complete response; DCR, disease control rate; ORR, objective response rate; PD, progressive disease; PR, partial response; SD, stable disease.

Data are presented as number of patients (%). P values for the comparison of ORR and DCR between the CIBP and non-CIBP groups were calculated using the χ² test.

Bold values indicate statistical significance (p < 0.05).

**Figure 2:**
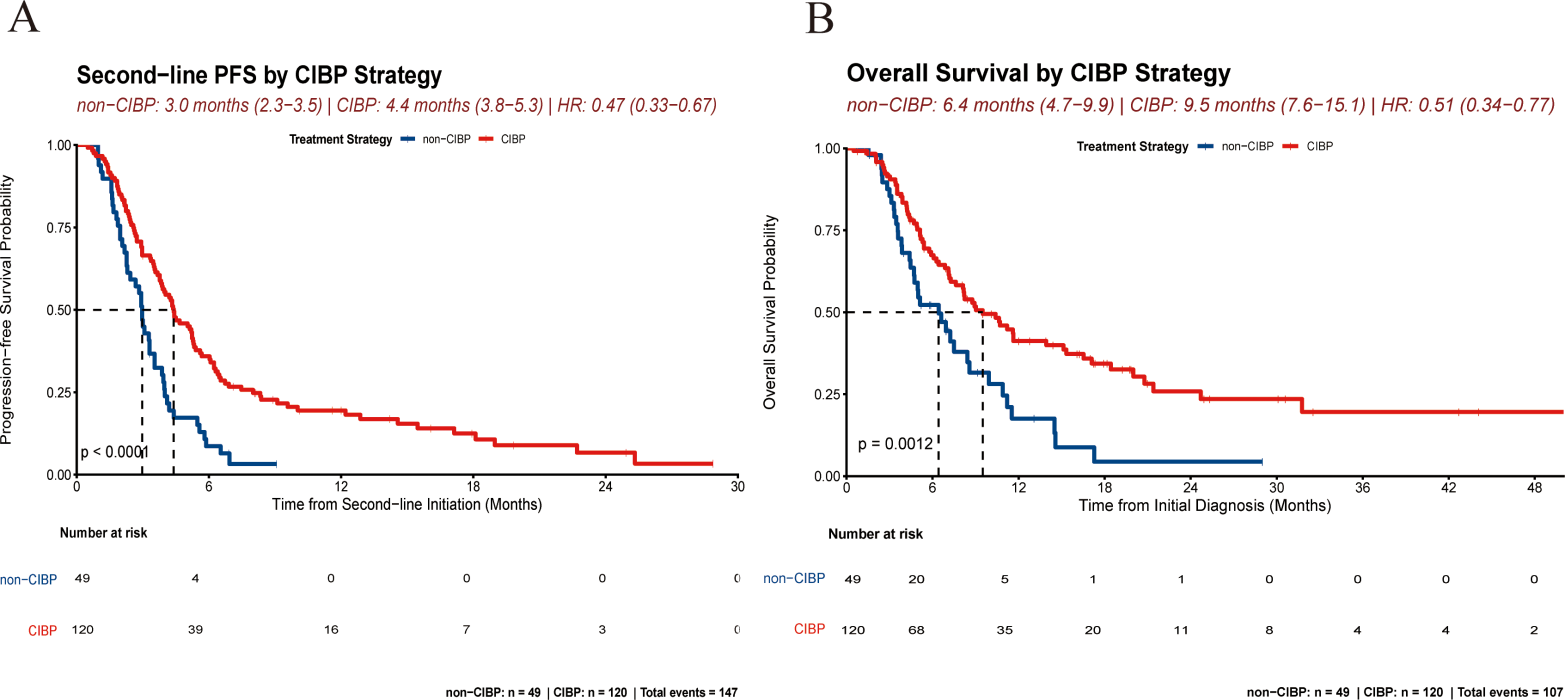
Kaplan-Meier curves for survival outcomes by second-line treatment strategy. **(A)** Second-line progression-free survival (PFS). The median PFS was 4.4 months (95% CI, 3.8-5.3) for the CIBP group versus 3.0 months (95% CI, 2.3-3.5) for the non-CIBP group (Hazard Ratio [HR] = 0.47; 95% CI, 0.33-0.67; log-rank p < 0.001). **(B)** Overall survival (OS). The median OS was 9.5 months (95% CI, 7.6-15.1) for the CIBP group versus 6.4 months (95% CI, 4.7-9.9) for the non-CIBP group (HR = 0.51; 95% CI, 0.34-0.77; log-rank p = 0.0012). CI, confidence interval; CIBP, continuing immune checkpoint inhibitor beyond progression; HR, hazard ratio. Survival differences between groups were compared using the log-rank test.

This enhanced disease control subsequently translated into a substantial survival benefit. Kaplan-Meier analysis revealed significantly prolonged survival outcomes for patients receiving the CIBP strategy (**Figure 2**). The median second-line progression-free survival (PFS) was 4.4 months (95% CI: 3.8-5.3) in the CIBP group, compared to 3.0 months (95% CI: 2.3-3.5) in the non-CIBP group (Log-rank p < 0.001; **Figure 2A**). The hazard ratio (HR) for disease progression or death was 0.47 (95% CI: 0.33-0.67) in favor of the CIBP strategy. Similarly, a significant overall survival (OS) advantage was observed (**Figure 2B**). The median OS was 9.5 months (95% CI: 7.6-15.1) for the CIBP group versus 6.4 months (95% CI: 4.7-9.9) for the non-CIBP group (Log-rank p = 0.0012), with an adjusted HR of 0.51 (95% CI: 0.34-0.77). For reference, the entire cohort (N = 169) had a median PFS of 3.9 months (95% CI: 3.4-4.4) and a median OS of 8.2 months (95% CI: 7.1-10.9) in the second-line setting (**Supplementary Figure 1**). Furthermore, within the CIBP group, patients with PD-L1 expression ≥1% showed a trend towards longer PFS (HR 0.64, 95% CI: 0.41-1.00) and OS (HR 0.68, 95% CI: 0.40-1.15) compared to those with PD-L1 <1%, although these differences were not statistically significant (**Supplementary Figure 2**).

### 3.3 Cox regression

To ascertain the independent prognostic value of the CIBP strategy, we performed univariable and multivariable Cox regression analyses. Variables with a p-value < 0.1 in the univariable analysis (including CIBP strategy, ECOG PS, number of metastatic sites, histology, liver metastasis, PD-L1 expression, response to first-line therapy, and PFS1 duration) were incorporated into the multivariable model. After adjusting for these potential confounders, the CIBP strategy remained an independent predictor for significantly prolonged PFS (adjusted Hazard Ratio [aHR] = 0.54, 95% CI: 0.35-0.81, p=0.003) and OS (aHR = 0.65, 95% CI: 0.42-0.99, p=0.044). Additionally, a good ECOG PS (0-1) was consistently identified as a strong independent factor favoring both PFS (aHR = 2.09, 95% CI: 1.44-3.04, p<0.001) and OS (aHR = 2.65, 95% CI: 1.77-3.97, p<0.001). Detailed results of the univariable and multivariable analyses are presented in **Table 3** and **4**, respectively.

**Table 3:** Univariate and multivariate cox regression analysis of factors associated with progression-free survival (PFS).

Variable	Univariate		Multivariate	
	HR (95% CI)	P value	HR (95% CI)	P value
Strategy (CIBP vs non-CIBP)	0.47 (0.33-0.67)	<0.001	0.54 (0.35-0.81)	0.003
Age (≥65 vs <65)	1.15 (0.83-1.60)	0.393		
Sex (Female vs Male)	1.07 (0.75-1.53)	0.697		
ECOG PS (≥2 vs 0-1)	0.41 (0.28-0.58)	<0.001	2.09 (1.44-3.04)	<0.001
Primary site (GEJ vs G)	0.80 (0.56-1.13)	0.204		
Histology (Non-intestinal vs Intestinal)	0.73 (0.52-1.01)	0.056	0.91 (0.64-1.30)	0.607
Metastatic sites (≥3 vs <3)	1.68 (1.19-2.37)	0.003	1.44 (0.99-2.08)	0.056
Liver metastasis (YES vs No)	1.43 (1.03-2.00)	0.035	1.23 (0.87-1.75)	0.235
Peritoneal metastasis (YES vs No)	1.00 (0.70-1.43)	0.999		
PD-L1 expression (≥1% vs <1%)	0.68 (0.47-1.00)	0.048	0.81 (0.53-1.26)	0.355
First-line response (SD/PD vs CR/PR)	1.67 (1.20-2.33)	0.002	1.26 (0.83-1.92)	0.272
PFS1 duration (≥6m vs <6m)	0.71 (0.52-0.99)	0.043	0.93 (0.65-1.33)	0.691

CI, confidence interval; CIBP, continuing immune checkpoint inhibitor beyond progression; CR, complete response; ECOG PS, Eastern Cooperative Oncology Group performance status; G, gastric; GEJ, gastroesophageal junction; HR, hazard ratio; PD, progressive disease; PFS, progression-free survival; PR, partial response; SD, stable disease.

Variables with a P value < 0.1 in the univariate analysis were included in the multivariate Cox proportional hazards model. Statistically significant P values (P < 0.05) are highlighted in bold.

**Table 4:** Univariate and multivariate cox regression analysis of factors associated with overall survival (OS).

Variable	Univariate		Multivariate	
	HR (95% CI)	P value	HR (95% CI)	P value
Strategy (CIBP vs non-CIBP)	0.51 (0.34-0.77)	0.001	0.65 (0.42-0.99)	0.044
Age (≥65 vs <65)	1.05 (0.72-1.54)	0.806		
Sex (Female vs Male)	1.32 (0.86-2.03)	0.199		
ECOG PS (≥2 vs 0-1)	0.34 (0.23-0.51)	<0.001	2.65 (1.77-3.97)	<0.001
Primary site (GEJ vs G)	0.76 (0.51-1.14)	0.186		
Histology (Non-intestinal vs Intestinal)	0.91 (0.62-1.34)	0.625		
Metastatic sites (≥3 vs <3)	1.25 (0.85-1.86)	0.257		
Liver metastasis (YES vs No)	1.17 (0.8-1.73)	0.419		
Peritoneal metastasis (YES vs No)	0.97 (0.64-1.46)	0.877		
PD-L1 expression (≥1% vs <1%)	0.8 (0.51-1.26)	0.339		
First-line response (SD/PD vs CR/PR)	1.82 (1.23-2.68)	0.003	1.45 (0.95-2.21)	0.082
PFS1 duration (≥6m vs <6m)	0.56 (0.38-0.82)	0.003	0.67 (0.45-1.01)	0.057

CI, confidence interval; CIBP, continuing immune checkpoint inhibitor beyond progression; CR, complete response; ECOG PS, Eastern Cooperative Oncology Group performance status; G, gastric; GEJ, gastroesophageal junction; HR, hazard ratio; PD, progressive disease; PFS, progression-free survival; PR, partial response; SD, stable disease.

Variables with a P value < 0.1 in the univariate analysis were included in the multivariate Cox proportional hazards model. Statistically significant P values (P < 0.05) are highlighted in bold.

### 3.4 Sensitivity analysis

To further validate the robustness of our primary findings, we conducted a propensity score matching (PSM) analysis to balance key prognostic baseline characteristics between the CIBP and non-CIBP groups. After 1:1 matching, 60 patients (30 pairs) were included with excellent balance achieved across all covariates (Standardized Mean Differences < 0.15 for all; **Supplementary Table 1**). Critically, the survival benefit of the CIBP strategy remained robust and statistically significant in this matched cohort. The CIBP group continued to demonstrate significantly superior PFS (matched HR = 0.35, 95% CI: 0.19-0.62, p < 0.001; **Supplementary Figure 3A**) and OS (matched HR = 0.42, 95% CI: 0.21-0.83, p = 0.011; **Supplementary Figure 3B**) compared to the non-CIBP group, thereby confirming the reliability of our primary analysis.

### 3.5 Subgroup analysis

**Figure 3:**
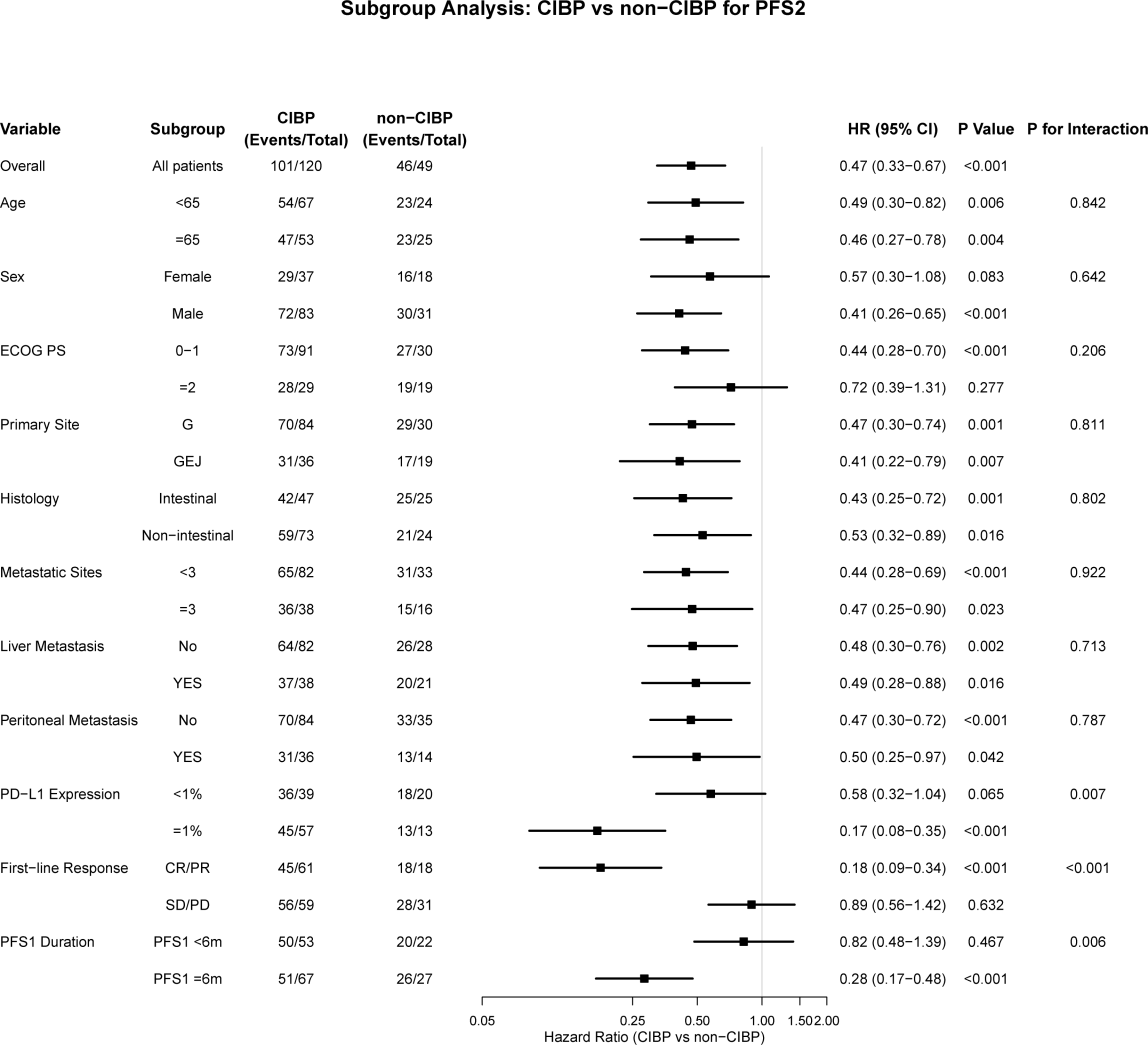
Subgroup analysis of progression-free survival. Forest plot of hazard ratios for progression-free survival (PFS) comparing the CIBP strategy with the non-CIBP strategy across patient subgroups. The size of the data markers corresponds to the sample size of each subgroup. A hazard ratio (HR) of less than 1.0 favors the CIBP group. The diamond at the bottom represents the overall HR and 95% confidence interval for the entire cohort. CI, confidence interval; CIBP, continuing immune checkpoint inhibitor beyond progression; CR, complete response; ECOG PS, Eastern Cooperative Oncology Group performance status; G, gastric; GEJ, gastroesophageal junction; HR, hazard ratio; PD, progressive disease; PFS, progression-free survival; PR, partial response; SD, stable disease.

**Figure 4:**
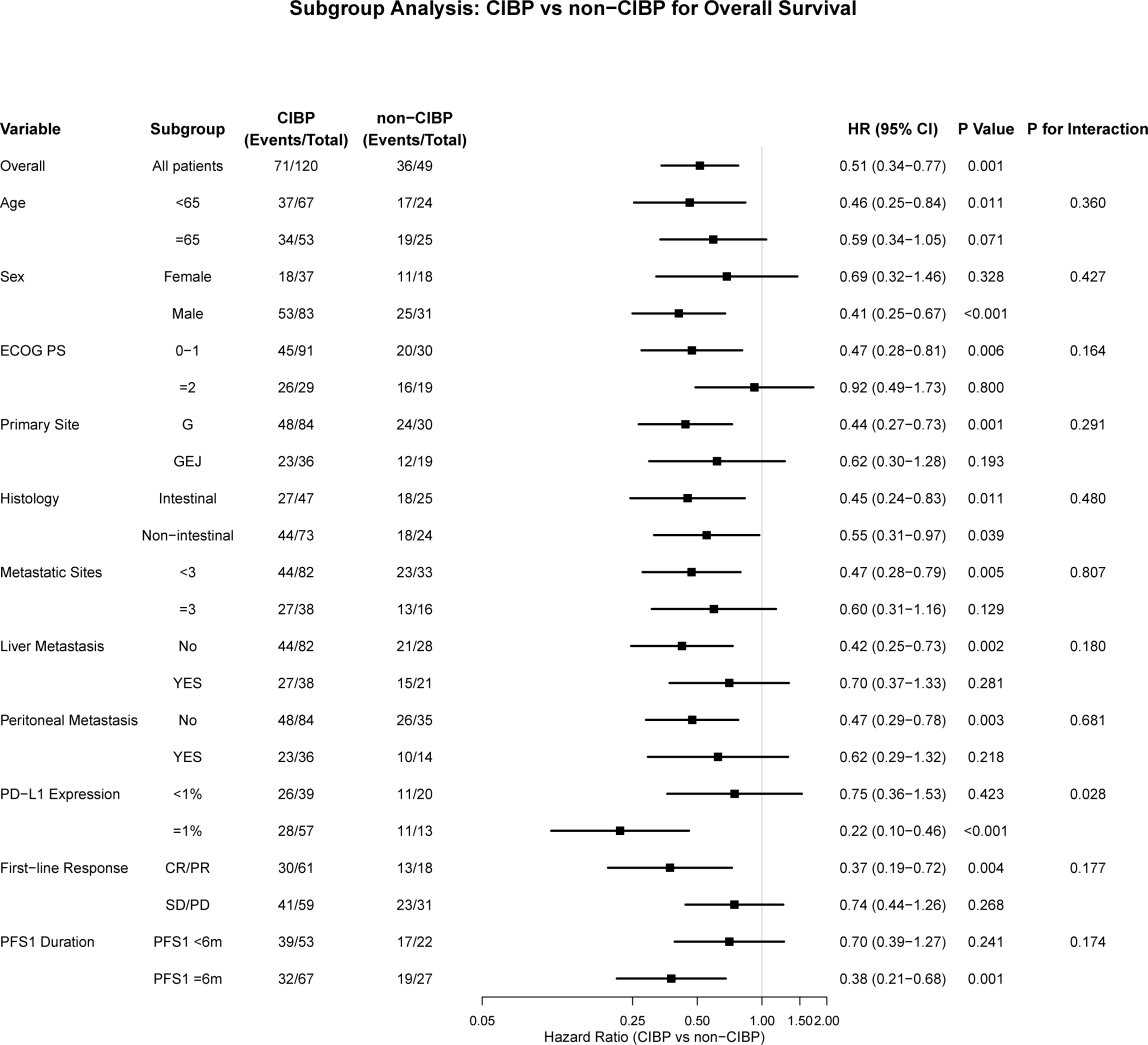
Subgroup analysis of overall survival. Forest plot of hazard ratios for overall survival (OS) comparing the CIBP strategy with the non-CIBP strategy across patient subgroups. The size of the data markers corresponds to the sample size of each subgroup. A hazard ratio (HR) of less than 1.0 favors the CIBP group. The diamond at the bottom represents the overall HR and 95% confidence interval for the entire cohort. CI, confidence interval; CIBP, continuing immune checkpoint inhibitor beyond progression; CR, complete response; ECOG PS, Eastern Cooperative Oncology Group performance status; G, gastric; GEJ, gastroesophageal junction; HR, hazard ratio; PD, progressive disease; OS, overall survival; PR, partial response; SD, stable disease.

Subgroup analyses demonstrated a consistent survival benefit favoring the CIBP strategy across all predefined subgroups (**Figure 3** for PFS, **Figure 4** for OS).

The magnitude of benefit was most substantial in several key cohorts. Patients with PD-L1 expression ≥1% exhibited markedly reduced risks of both progression (HR = 0.17, 95% CI: 0.08-0.35) and death (HR = 0.22, 95% CI: 0.10-0.46), with significant interaction terms for both PFS (P for interaction = 0.007) and OS. Furthermore, the treatment effect was strongly associated with the efficacy of first-line therapy: patients who achieved an objective response (CR/PR) to first-line treatment showed significantly improved PFS (HR = 0.58, 95% CI: 0.32-1.04), while those with a durable clinical benefit from first-line therapy (PFS1 ≥6 months) experienced profound risk reductions in both PFS (HR = 0.28, 95% CI: 0.17-0.49) and OS (HR = 0.38, 95% CI: 0.21-0.68), with a significant interaction for PFS (P for interaction = 0.006). The treatment effect was consistent across all other subgroups, as indicated by non-significant interaction tests.

### 3.6 Safety

The safety profile was evaluated in all 169 patients. As summarized in **Table 5**, the overall incidence of adverse events was high and comparable between the CIBP and non-CIBP groups (94.2% vs 95.9%), which was expected in this heavily pre-treated population. The spectrum and incidence of toxicities were consistent with the known safety profiles of the constituent drugs. Gastrointestinal toxicities (e.g., nausea, vomiting) and hematologic toxicities (e.g., anemia, leukopenia) were the most common adverse events, and their frequencies were balanced between the two groups, aligning with the anticipated effects of chemotherapy. The overall incidence and severity of immune-related adverse events (irAEs) in the CIBP group were manageable and consistent with those reported in pivotal trials of immune checkpoint inhibitors. Critically, no new or unexpected safety signals were identified in the CIBP group. Dose modifications, including treatment delays, dose reductions, or permanent discontinuations, due to adverse events occurred in 21.3% of the overall population (17.5% in the CIBP group vs 30.6% in the non-CIBP group).

**Table 5:** Summary of treatment-emergent adverse events.

Adverse event	Total (n=169)		CIBP (n=120)		Non-CIBP (n=49)	
	Any grade	G3-4	Any grade	G3-4	Any grade	G3-4
Any adverse event	160 (94.7)		113 (94.2)		47 (95.9)	
Dose modification^*^	36 (21.3)		21 (17.5)		15 (30.6)	
GI toxicity						
Nausea	27 (16.0)	-	19 (15.8)	-	8 (16.3)	-
Vomiting	19 (11.2)	1 (0.6)	11 (9.2)	-	8 (16.3)	1 (2.0)
Diarrhea	6 (3.6)	1 (0.6)	3 (2.5)	-	3 (6.1)	1 (2.0)
Hematologic toxicity						
Anemia	70 (41.4)	8 (4.7)	48 (40.0)	5 (4.2)	22 (44.9)	3 (6.1)
Leukopenia	67 (39.6)	11 (6.5)	49 (40.8)	10 (8.3)	18 (36.7)	1 (2.0)
Neutropenia	28 (16.6)	9 (5.3)	22 (18.3)	9 (7.5)	6 (12.2)	-
Thrombocytopenia	44 (26.0)	7 (4.1)	29 (24.2)	4 (3.3)	15 (30.6)	3 (6.1)
Immune-related adverse event						
Hyperthyroidism	2 (1.2)	1 (0.6)	-	-	2 (4.1)	1 (2.0)
Hypothyroidism	47 (27.8)	2 (1.2)	36 (30.0)	2 (1.7)	11 (22.4)	-
Pneumonitis	5 (3.0)	1 (0.6)	-	-	5 (10.2)	1 (2.0)
Myocarditis	3 (1.8)	2 (1.2)	1 (0.8)	-	2 (4.1)	2 (4.1)
Neuropathy	22 (13.0)	2 (1.2)	17 (14.2)	1 (0.8)	5 (10.2)	1 (2.0)
Rash	2 (1.2)	1 (0.6)	2 (1.7)	1 (0.8)	-	-
Biochemical abnormalities						
ALT	21 (12.4)	6 (3.6)	14 (11.7)	4 (3.3)	7 (14.3)	2 (4.1)
AST	19 (11.2)	5 (3.0)	13 (10.8)	3 (2.5)	6 (12.2)	2 (4.1)
Cr	5 (3.0)	-	5 (4.2)	-	-	-
TBIL	11 (6.5)	2 (1.2)	8 (6.7)	2 (1.7)	3 (6.1)	-
ALB	19 (11.2)	1 (0.6)	13 (10.8)	1 (0.8)	6 (12.2)	-

ALB, albumin; ALT, alanine aminotransferase; AST, aspartate aminotransferase; CIBP, continuing immune checkpoint inhibitor beyond progression; Cr, creatinine; TBIL, total bilirubin.

Data are presented as number of patients (%). Dose modification* includes treatment delay, dose reduction, or permanent discontinuation due to an adverse event. Adverse events were graded according to the National Cancer Institute Common Terminology Criteria for Adverse Events (CTCAE), version 5.0.

## 4 Discussion

### 4.1 Summary of key findings and study significance

Our real-world study demonstrates that continuing immune checkpoint inhibitor (ICI) therapy beyond progression (CIBP) in patients with advanced gastric or gastroesophageal junction cancer (GC/GEJC) who progressed after first-line immuno-chemotherapy was associated with significantly improved progression-free survival (PFS) and overall survival (OS), with a manageable safety profile. Despite the established role of ICIs in the first-line setting for advanced GC, as evidenced by landmark trials (5–8), the optimal therapeutic strategy after disease progression—particularly whether to continue ICI therapy—remains controversial and is not addressed in current clinical guidelines. High-quality real-world evidence in this area is scarce (9). Beyond ICI−based strategies, standard second−line options include ramucirumab plus paclitaxel; for HER2−positive patients who progress on prior trastuzumab, antibody–drug conjugates (ADCs) such as trastuzumab deruxtecan and disitamab vedotin have shown efficacy; and zolbetuximab targeting CLDN18.2 has emerged as a first−line option for CLDN18.2−positive tumors (2). Moreover, accumulating real−world data support the feasibility of immunotherapy rechallenge or continuation beyond progression in gastric cancer (18). Therefore, this study was designed to provide empirical insights into this critical clinical dilemma by comparing the efficacy and safety of CIBP versus non-CIBP strategies.

### 4.2 Validation and generalizability of the CIBP benefit

The survival benefit observed with the CIBP strategy was consistent across multiple analytical methods. Initial separation of the Kaplan-Meier curves for PFS and OS was observed, with univariable analysis indicating a reduced risk of progression or death. To account for potential confounding, a multivariable Cox model was applied. After adjustment for prognostic covariates, the CIBP strategy remained associated with improved PFS (aHR = 0.54) and OS (aHR = 0.65). To further strengthen our findings, a sensitivity analysis using propensity score matching (PSM) was conducted to emulate a randomized study design (10), which also showed a survival benefit for the CIBP group (matched PFS HR = 0.35; matched OS HR = 0.42).

The generalizability of these findings is supported by the composition of our real-world cohort. Our study included patients often underrepresented in clinical trials, such as those with ECOG PS 2, HER2-positive status, and a high metastatic burden. Despite this heterogeneity, the association between CIBP and survival outcomes remained after statistical adjustment for these and other baseline characteristics. This suggests that the potential benefit of continuing ICI therapy may be applicable to a clinically diverse patient population in real-world settings.

### 4.3 Biological plausibility and predictive biomarkers

The Kaplan-Meier curve for overall survival in the CIBP group exhibited a protracted tail, a pattern not observed in the non-CIBP group. This divergence aligns with the recognized delayed separation of survival curves in immunotherapy, which reflects its distinct mode of action centered on reinvigorating a sustained anti-tumor immune response, rather than the direct cytotoxic effects of chemotherapy (11). This observed long-tail signature provides a clinical rationale for sustaining immune modulation beyond initial progression.

The efficacy of the CIBP strategy was most pronounced in specific patient subsets. Patients who achieved an objective response (ORR) or experienced a progression-free survival of ≥6 months (PFS1 ≥6m) during first-line immuno-chemotherapy derived a more substantial survival benefit from CIBP. This finding strongly suggests that an established response to prior ICI therapy may identify patients with a tumor microenvironment primed for ongoing PD-1/PD-L1 blockade (12). Furthermore, PD-L1 expression emerged as a critical effect modifier. The significantly reduced hazards for PFS and OS observed in patients with PD-L1 CPS ≥1%, supported by significant interaction terms, reinforce the biological plausibility of our findings and are consistent with the foundational role of PD-L1 as a key predictive biomarker for ICI efficacy established in landmark trials (5). Conversely, the impact of other established biomarkers in GC, such as HER2 and mismatch repair (MMR) status, on CIBP benefit could not be definitively assessed in this cohort due to sample size constraints and limited subgroup numbers.

Beyond tumor-intrinsic features, our data underscore the paramount importance of host factors. A good ECOG performance status (0-1) was consistently identified as one of the strongest independent prognostic factors for superior survival. This is highly congruent with extensive literature across advanced cancers, which positions ECOG PS as a cornerstone of prognosis, reflecting the patient’s functional and immunological reserve necessary to mount and sustain an effective antitumor response (13).

Conversely, the survival benefit associated with the CIBP strategy appeared attenuated in patients with metastases to specific sites, notably the liver (HR = 0.60) and peritoneum (HR = 0.70). This converging pattern is biologically insightful. Both liver and peritoneal metastases are recognized as entities with particularly poor prognosis in gastric cancer (14). More importantly, they are increasingly understood to create highly immunosuppressive niches. The liver can systemically sequester and inactivate T cells, while the peritoneal cavity may pose a physical barrier and be enriched with immunosuppressive cells (15). Specifically, in gastric cancer peritoneal metastasis, the limited response to systemic therapies may be linked to the phenomenon where resident macrophages are hijacked and polarized towards a pro-tumor M2-like phenotype, which fosters an immunosuppressive microenvironment (16). The numerically reduced efficacy of CIBP in these subgroups suggests that the presence of such immunosuppressive metastases might partially counteract the systemic immune reactivation intended by continued ICI therapy. This hypothesis warrants future investigation into combination strategies designed to reprogram these hostile microenvironments.

The potential biological mechanism underlying the benefit of continuing ICI beyond progression may involve sustained reinvigoration of pre−existing tumor−specific T−cell responses. Even after radiographic progression, a subset of tumor microenvironments may retain sensitivity to PD−1/PD−L1 blockade, and continued ICI could prevent or delay T−cell exhaustion, as suggested by the protracted tail of the survival curve in our CIBP group (11, 12).

Regarding differences among PD−1 inhibitors, our sample size was not sufficient to perform reliable subgroup analyses for individual agents. Nevertheless, PD−1 inhibitors differ in molecular structure, Fc region functionality, binding epitopes, and pharmacokinetic properties. For example, Fc−competent antibodies may induce antibody−dependent cellular cytotoxicity (ADCC) affecting T−cell homeostasis, whereas Fc−silent antibodies might have a different immunological profile. Whether these differences influence the efficacy of CIBP remains unknown and warrants prospective investigation.

### 4.4 Safety evaluation of continuing ICI beyond progression

The therapeutic strategy of continuing ICI beyond progression is gaining clinical interest, with supporting evidence emerging not only from other malignancies like non-small cell lung cancer (17) but also from within the field of gastric cancer itself. A growing body of retrospective real-world studies, including a recent investigation specifically examining immunotherapy rechallenge in advanced GC, has begun to outline the potential feasibility and efficacy of this approach (18). Our findings provide additional real-world data that align with and help to characterize this emerging treatment option.

However, translating this concept into routine practice for patients who have progressed on first-line therapy necessitates a thorough evaluation of safety, particularly given this population’s often declining performance status and accumulated toxicities. Our comprehensive safety analysis offers critical insights on this front. The overall incidence of adverse events was comparable between the CIBP and non-CIBP groups. The toxicity profile in the CIBP group aligned with the expected patterns of the constituent drugs, with no new or unexpected safety signals identified.

Particularly noteworthy is the analysis of immune-related adverse events (irAEs). The incidence and severity of irAEs in the CIBP group were manageable and did not suggest a synergistic or cumulative toxicity profile. This observation is clinically reassuring, as it indicates that continuing ICI therapy in this pre-treated population was not associated with an exacerbation of immune-related toxicity beyond what is typically expected. Consequently, our data suggest that with appropriate monitoring and management, the CIBP strategy does not impose an unmanageable safety burden, thereby mitigating a major practical reservation towards its clinical adoption.

### 4.5 Limitations and future perspectives

Despite the robust findings observed in our analysis, several limitations inherent to its design must be acknowledged. First, the retrospective, single-center nature of this study is susceptible to potential selection bias and unmeasured confounding factors, despite our efforts to adjust for known prognostic variables using multivariable Cox regression and propensity score matching. Second, the sample size, though substantial for a real-world study on this specific clinical question, remains limited, particularly for subgroup analyses and for comparing the efficacy of different ICI-based combination regimens within the CIBP group. This precludes definitive conclusions regarding the optimal partner for ICI in the second-line setting. Third, the heterogeneity in second-line treatment regimens within both the CIBP and non-CIBP groups, while reflecting real-world practice, introduces another layer of complexity.

These limitations, however, help to chart a course for future research. The promising efficacy and manageable safety profile of the CIBP strategy identified in our study strongly advocate for its validation in larger, multi-center, prospective cohorts. Such studies would be crucial to confirm our findings and minimize biases. Moreover, our data underscore that not all patients derive equal benefit. Future investigations should therefore prioritize the development of more precise patient selection strategies. This includes validating the predictive value of clinical indicators such as response to first-line therapy and ECOG PS, and further exploring the role of PD-L1 expression in this specific setting. Ultimately, the pursuit of more sophisticated biomarkers—such as tumor mutational burden, microbiome signatures, or dynamic changes in the T-cell repertoire—is warranted to better identify the ideal candidates who would derive the maximum survival advantage from continuing immune checkpoint inhibition beyond initial progression.

## 5 Conclusion

In this real-world study, continuing immune checkpoint inhibitor therapy beyond progression was associated with significantly superior survival outcomes compared to non-ICI regimens in patients with advanced gastric cancer. This benefit was consistent after multivariable adjustment and propensity score matching, with a manageable safety profile. Our findings support CIBP as a valid second-line strategy for this patient population.

## Data Availability

The data analyzed in this study is subject to the following licenses/restrictions: The data are not publicly available due to patient privacy and confidentiality restrictions. Access is restricted but can be granted upon reasonable request to the corresponding author for research purposes, subject to institutional review. Requests to access these datasets should be directed to YW, onco_yunmei@163.com.

## Supplementary material

Supplementary Figure 1 Survival outcomes for the entire second-line cohort. Kaplan-Meier curves for **(A)** progression-free survival (PFS) and **(B)** overall survival (OS) of all eligible patients who received second-line therapy (N = 169). The median PFS was 3.9 months (95% CI, 3.4-4.4). The median OS was 8.2 months (95% CI, 7.1-10.9).

Supplementary Figure 2 Survival outcomes by PD-L1 expression within the CIBP group. Kaplan-Meier curves for **(A)** progression-free survival (PFS) and **(B)** overall survival (OS) comparing patients with PD-L1 expression ≥1% to those with PD-L1 <1%, within the cohort that continued ICI beyond progression (CIBP group). Hazard ratios (HRs) with 95% confidence intervals (CIs) are shown. The differences were not statistically significant.

Supplementary Figure 3 Sensitivity analysis: survival outcomes in the propensity score-matched cohort. Kaplan-Meier curves for **(A)** progression-free survival (PFS) and **(B)** overall survival (OS) comparing the CIBP and non-CIBP groups in the propensity score-matched cohort (30 pairs, n=60). The CIBP strategy continued to show significantly superior PFS (matched HR = 0.35; 95% CI, 0.19-0.62; p < 0.001) and OS (matched HR = 0.42; 95% CI, 0.21-0.83; p = 0.011) compared to the non-CIBP strategy.
