# Supplementary figures and images for: Continuing immune checkpoint inhibitors beyond progression versus switching to non-ICI therapy in advanced gastric cancer: a real-world study
Source: Front Oncol. 2026 Jun 3;16:1798205. doi: 10.3389/fonc.2026.1798205 (PMC13272025; doi:10.3389/fonc.2026.1798205)

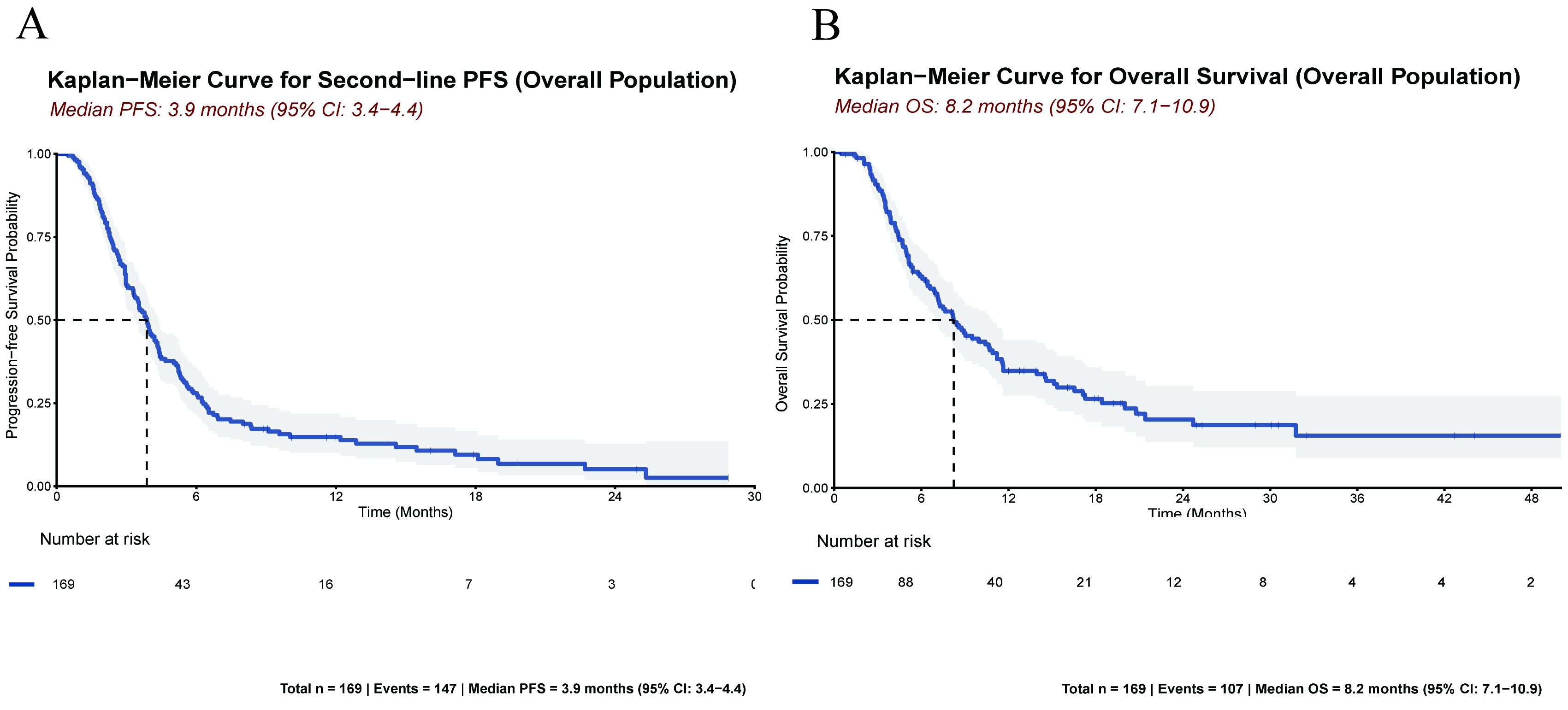

Supplement: Supplementary Figure 1 — Survival outcomes for the entire second-line cohort. Kaplan-Meier curves for (A) progression-free survival (PFS) and (B) overall survival (OS) of all eligible patients who received second-line therapy (N = 169). The median PFS was 3.9 months (95% CI, 3.4-4.4). The median OS was 8.2 months (95% CI, 7.1-10.9). [file Image1.tif]

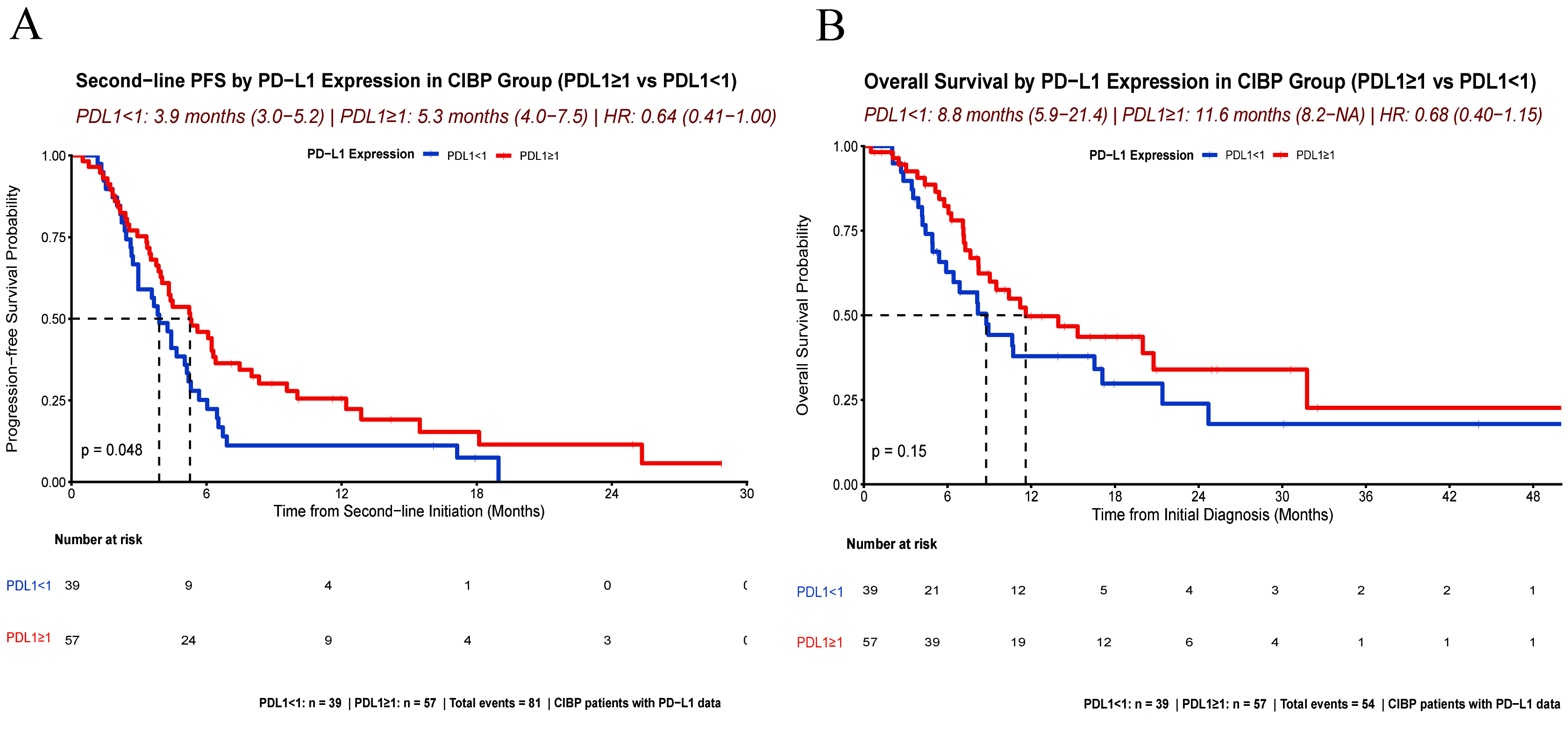

Supplement: Supplementary Figure 2 — Survival outcomes by PD-L1 expression within the CIBP group. Kaplan-Meier curves for (A) progression-free survival (PFS) and (B) overall survival (OS) comparing patients with PD-L1 expression ≥1% to those with PD-L1 <1%, within the cohort that continued ICI beyond progression (CIBP group). Hazard ratios (HRs) with 95% confidence intervals (CIs) are shown. The differences were not statistically significant. [file Image2.tif]

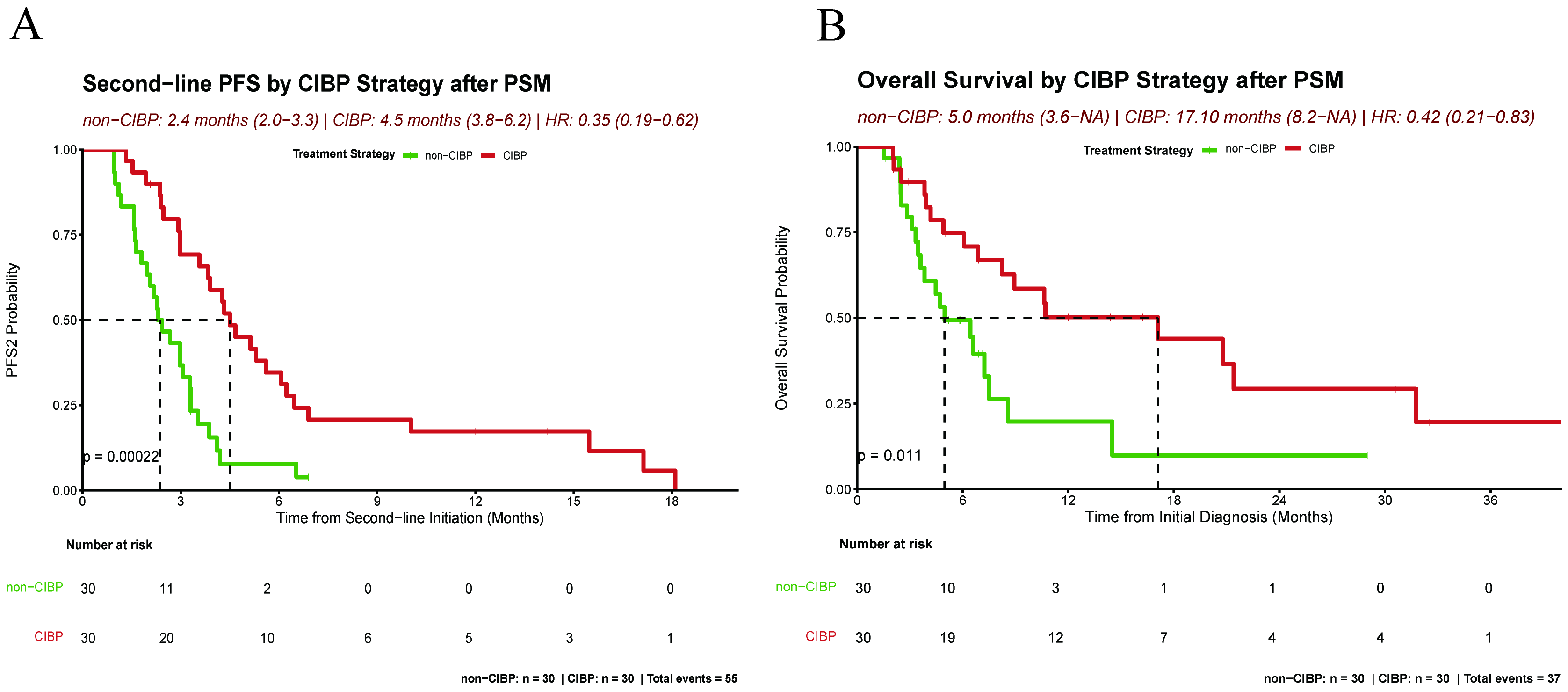

Supplement: Supplementary Figure 3 — Sensitivity analysis: survival outcomes in the propensity score-matched cohort. Kaplan-Meier curves for (A) progression-free survival (PFS) and (B) overall survival (OS) comparing the CIBP and non-CIBP groups in the propensity score-matched cohort (30 pairs, n=60). The CIBP strategy continued to show significantly superior PFS (matched HR = 0.35; 95% CI, 0.19-0.62; p < 0.001) and OS (matched HR = 0.42; 95% CI, 0.21-0.83; p = 0.011) compared to the non-CIBP strategy. [file Image3.tif]

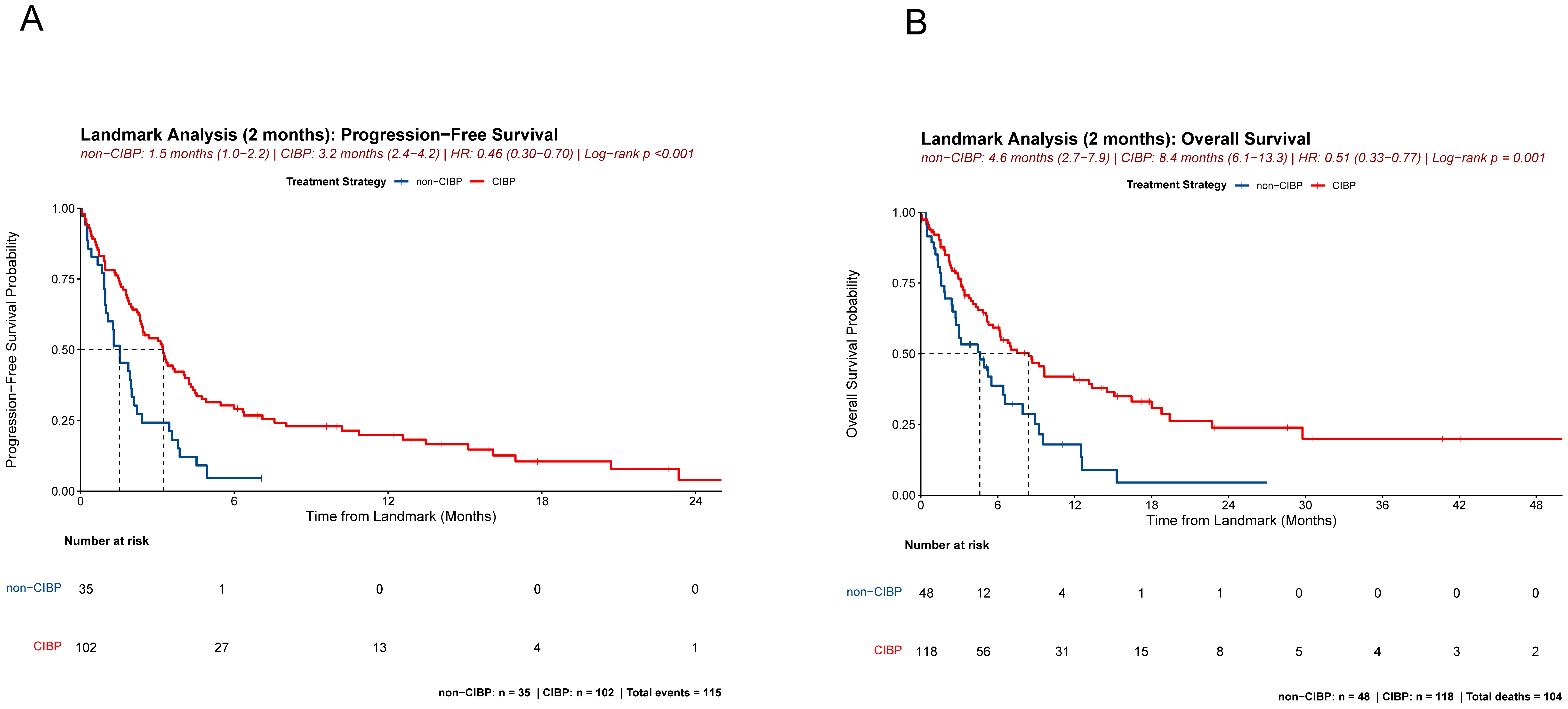

Supplement: Supplementary file 4 [file Image4.tif]
