# Supplementary material for: Continuing immune checkpoint inhibitors beyond progression versus switching to non-ICI therapy in advanced gastric cancer: a real-world study
Source: Front Oncol. 2026 Jun 3;16:1798205. doi: 10.3389/fonc.2026.1798205 (PMC13272025; doi:10.3389/fonc.2026.1798205)
Supplement: Supplementary file 5 [file Table1.docx]

**Supplementary Table S1. Baseline Characteristics of the Propensity Score-Matched Cohort.**

| **Characteristic** | **Overall (N = 60)** | **CIBP (N = 30)** | **non-CIBP (N = 30)** | **SMD** |
| --- | --- | --- | --- | --- |
| Age, ≥65 years | 31 (51.7%) | 15 (50%) | 16 (53.3%) | 0.067 |
| ECOG PS, ≥2 | 18 (30%) | 10 (33.3%) | 8 (26.7%) | 0.146 |
| Peritoneal metastasis | 19 (31.7%) | 8 (26.7%) | 11 (36.7%) | 0.216 |
| Metastatic sites, ≥3 | 23 (38.3%) | 11 (36.7%) | 12 (40%) | 0.069 |
| Histology, Non-intestinal | 27 (45%) | 12 (40%) | 15 (50%) | 0.202 |
| PD-L1 expression, ≥1% | 28 (46.7%) | 15 (50%) | 13 (43.3%) | 0.134 |
| PFS1 > 6 months | 34 (56.7%) | 17 (56.7%) | 17 (56.7%) | <0.001 |

**Abbreviations:** CIBP, continuing immune checkpoint inhibitor beyond progression; ECOG PS, Eastern Cooperative Oncology Group performance status; PFS, progression-free survival; SMD, standardized mean difference.

**Notes:** Data are presented as number of patients (n) and percentage (%). The propensity score-matched cohort consisted of 60 patients (30 pairs). Balance between the CIBP and non-CIBP groups after matching was assessed using the standardized mean difference (SMD), with an SMD < 0.15 indicating a well-balanced variable.
