# Supplementary material for: Continuing immune checkpoint inhibitors beyond progression versus switching to non-ICI therapy in advanced gastric cancer: a real-world study
Source: Front Oncol. 2026 Jun 3;16:1798205. doi: 10.3389/fonc.2026.1798205 (PMC13272025; doi:10.3389/fonc.2026.1798205)
Supplement: Supplementary file 6 [file Table2.docx]

**Supplementary Methods** – Landmark analysis at 2 months

To address potential immortal time bias, we performed a landmark analysis at 2 months after the start of second‑line therapy. Patients who experienced an event (disease progression or death for progression‑free survival [PFS]; death for overall survival [OS]) within the first 2 months were excluded. For the remaining patients, time zero was reset to 2 months, and the follow‑time was recalculated as the original survival time minus 2 months. Patients who were censored within the first 2 months remained in the analysis but contributed zero follow‑up time from the landmark. Kaplan‑Meier curves and Cox proportional hazards models were then applied to compare the CIBP and non‑CIBP groups, with the latter serving as the reference. All other analytical settings were identical to those described in the main Methods section.

**Supplementary Table S2. Landmark analysis at 2 months for progression‑free survival and overall survival**

| **Endpoint** | **Group comparison** | **No. of patients** | **Hazard ratio (95% CI)** | **P value** |
| --- | --- | --- | --- | --- |
| PFS | CIBP vs. non‑CIBP | 102 vs. 35 | 0.46 (0.30–0.70) | < 0.001 |
| OS | CIBP vs. non‑CIBP | 118 vs. 48 | 0.51 (0.33–0.77) | 0.001 |

Abbreviations: CIBP, continuing immune checkpoint inhibitor beyond progression; PFS, progression‑free survival; OS, overall survival; CI, confidence interval.

Notes: Patients who experienced an event (disease progression or death for PFS; death for OS) within the first 2 months of second‑line therapy were excluded. Time zero was reset to 2 months.
